# Change of Willingness to Accept COVID-19 Vaccine and Reasons of Vaccine Hesitancy of Working People at Different Waves of Local Epidemic in Hong Kong, China: Repeated Cross-Sectional Surveys

**DOI:** 10.3390/vaccines9010062

**Published:** 2021-01-18

**Authors:** Kailu Wang, Eliza Lai-Yi Wong, Kin-Fai Ho, Annie Wai-Ling Cheung, Peter Sen-Yung Yau, Dong Dong, Samuel Yeung-Shan Wong, Eng-Kiong Yeoh

**Affiliations:** Centre for Health Systems and Policy Research, JC School of Public Health and Primary Care, Faculty of Medicine, The Chinese University of Hong Kong, Hong Kong, China; kailuwang@cuhk.edu.hk (K.W.); kfho@cuhk.edu.hk (K.-F.H.); anniewlcheung@cuhk.edu.hk (A.W.-L.C.); b144754@cuhk.edu.hk (P.S.-Y.Y.); dongdong@cuhk.edu.hk (D.D.); yeungshanwong@cuhk.edu.hk (S.Y.-S.W.); yeoh_ek@cuhk.edu.hk (E.-K.Y.)

**Keywords:** COVID-19 vaccine, vaccine acceptance, vaccine hesitancy, vaccine safety, health behaviour, occupation

## Abstract

Vaccine hesitancy is among the major threats to the effectiveness of vaccination programmes. This study aimed to report the trend in response to willingness to accept the COVID-19 vaccine between two waves of the local epidemic and examine differences among occupations. Two cross-sectional surveys were conducted online during the first wave (February) and third wave (August to September) of the local epidemic in 2020. Acceptance of the COVID-19 vaccine was measured along with personal protection behaviours and occupations. A total of 2047 participants provided valid responses. The willingness to accept the COVID-19 vaccine among the participants was lower in the third wave (34.8%) than the first wave (44.2%). There were more concerns over vaccine safety in the third wave. Clerical/service/sales workers were less likely to accept the vaccine (adjusted odds ratio: 0.62, 95% confidence interval: 0.43–0.91). A high-level compliance of facemask wearing was found, and more people maintained social distancing and used alcohol hand rub in the third wave. Decreasing willingness to accept the COVID-19 vaccine may be associated with increasing concerns about vaccine safety and growing compliance of personal protection behaviours. The rush of vaccine development with higher risks of safety issues may jeopardize the public’s trust and lower uptake rates. Education and favourable policy should be provided to the general working population for the vaccination, especially for those who are not professional and are frequently exposed to crowds.

## 1. Introduction

As the daily number of confirmed coronavirus disease 2019 (COVID-19) cases increased worldwide throughout the year of 2020, global disease burden posed by the pandemic is heavy [1,2], while there is no specific medical treatment of COVID-19 to date [3]. Therefore, the development and implementation of COVID-19 vaccines becomes crucial to the prevention and elimination of the disease [4]. By the end of October 2020, there were at least 44 vaccine candidates being tested in clinical trial and nine of them in phase 3 trials globally [5,6]. In addition to the efficacy of the vaccines, their uptake rate is also important to the effectiveness of preventing the spread of COVID-19 in future [7,8], as it needs to achieve certain levels to create herd immunity among the population [9].

However, it has been reported that vaccine refusal and hesitancy have been increasing in recent years [10]. This vaccine hesitancy, which could lead to refusal or delay of the vaccination, may eventually cause a reduction in coverage rate of the vaccine and affect its effectiveness [11,12,13]. Vaccine hesitancy was also listed among the top ten global health threats by the World Health Organization (WHO) [10]. Thus, there is a need to ascertain the level of willingness to accept the COVID-19 vaccine when the potential one becomes available.

A few studies have been conducted worldwide to identify the factors associated with COVID-19 vaccine acceptance among the general population. Recent studies in US, Canada and Australia found that age, sex, education level and ethnicity were associated with the intention to uptake the COVID-19 vaccine [14,15,16]. A study in mainland China [17] and a study in Malaysia [18] identified that perceived risks of infection and previous uptake of influenza vaccination could also affect COVID-19 vaccine acceptance. In addition to the general population, studies on vaccination acceptance of health care workers are most common in current literature, as they are considered to have the highest risks of infection [19,20]. However, there are limited studies on the vaccine acceptance among other occupations which also have a high chance of infection under the pandemic, such as retail or restaurant workers with direct exposure to customers [21]. Transmission of the disease in workplaces other than healthcare settings was substantial [22,23]. Therefore, it is also necessary to understand vaccination acceptance among different occupation groups.

In this regard, this study aimed to explore the willingness to accept the COVID-19 vaccine and identify reasons for refusal and hesitancy and their trends between two waves of local epidemic at different time points, which is helpful to estimate future uptake rate when the vaccine is available and to design promotional strategies before the launch of vaccination programmes. This study will also examine the association of this willingness with occupation types.

## 2. Materials and Methods

This study comprised two cross-sectional online surveys among the working population in Hong Kong (HK), China. This first survey was conducted from 17 to 27 February 2020 when the “first wave” of the local COVID-19 epidemic occurred and before declaration of the pandemic by WHO on 14 March 2020 [24]; the second survey was conducted from 24 August to 7 September 2020 when the “third wave” of the local epidemic was coming to an end [25,26]. Ethics approval of this study was obtained from the Survey and Behavioural Research Ethics Committee of The Chinese University of Hong Kong.

### 2.1. Study Sample and Data Collection

This study targeted the working population in HK, which includes nine occupation groups as categorized by the government, namely, (1) professionals, (2) managers and administrators, (3) associate professionals, (4) clerical support workers, (5) service and sales workers, (6) craft and related workers, (7) plant and machine operators and assemblers, (8) elementary occupations and (9) others [27]. Those aged 18 or above who were either employed or self-employed, working on a full-time or part-time basis and understood Chinese were eligible for the study. Those who were retired, housewives or students were excluded.

In the first survey, the online questionnaire was made available to the target population through email and multiple social networks, as this is considered to be a safer way for both participants and research team members to avoid face-to-face contact and reduce risk of infection. The second survey was delegated to an online survey company for data collection to improve the sample representativeness. Questionnaires of both surveys were self-administered and in Chinese. An information sheet including the details of the study was available at the beginning of both surveys. Electronic consent was obtained from the participants before each survey. Data collected from the surveys were retrieved from an online database and protected by passwords.

### 2.2. Measurements

The questionnaire for both surveys was developed with reference to a related study in personal protection practice to prevent infectious diseases [28] and WHO guidelines for COVID-19 prevention in the workplace [29]. It contained three major aspects: (1) socio-demographic characteristics, including age, sex, occupation, education level, and marital status; (2) frequency of personal protection behaviours in the past seven days, including “use of alcohol-based handrub when outside”, “wearing a surgical facemask when outside” and social distancing (combining “avoiding leaving home” and “avoiding contact with neighbours/relatives/friends”), along with a three-point scale to indicate that behaviours were “never”, “sometimes” or “usually/always” performed in the past seven days; and (3) previous influenza vaccination behaviours and willingness to accept a potential COVID-19 vaccine, as well as reasons for refusal or hesitancy. The willingness to accept the COVID-19 vaccine was determined by the following: “if a COVID-19 vaccine is available now, whether or not will you choose to accept it”. The responses consisted of three categories, namely, “Yes (accept)”, “No (refuse)” and “Undecided”. To identify the reasons for vaccine refusal and hesitancy, the respondents were asked the following question: “What is/are the reasons that you are not willing to have vaccination or have not decided yet?”, and they could choose responses from one or more options including “do not trust the effectiveness of the vaccine”, “do not think it is necessary”, “no time for vaccination”, “vaccine is susceptible to be infected with COVID-19”, “serious adverse side effects are common in vaccine”, “unforeseen side effect of vaccine has not been fully discovered” and “vaccine safety is controversial”. The latter three options were considered as “worries about safety of vaccine” in the analysis.

To reduce the complexity of finding interpretations, the occupation types were re-grouped through the analysis. Thus, clerical support workers and service/sales workers were combined and named as “clerical/service/sales workers”; craft and related workers, plant and machine operators and assemblers, elementary occupations and others were combined as “blue-collar workers”. As for frequencies of personal protection behaviours, there were too few participants who never performed such behaviours, so “never” and “sometimes” were re-grouped as “never/sometimes” in the analysis.

### 2.3. Statistical Analysis

Descriptive analysis was performed for the socio-demographic characteristics of the two surveys. The frequency of personal protection behaviours and willingness to accept the COVID-19 vaccine among participants were summarized using cross-tabulation. Chi-square tests were performed to identify their differences. Those with missing values in personal protection behaviours and vaccine willingness were excluded from the analysis. In order to make the behaviours and vaccine willingness in these two surveys comparable, the sample of the first survey was standardized using a direct method based on the age, sex and occupational distribution of the sample in the second survey [30]. Following this, multiple multinomial logistic regression was applied to examine the association between occupation and willingness to accept the COVID-19 vaccine in the two different rounds of survey, with adjustment for age, sex, marriage, chronic conditions and previous influenza vaccination behaviours. Multiple logistic regressions were also performed to determine the difference in personal protection behaviours between these two rounds of survey and among different occupation groups with adjustment for the aforementioned covariates. Reasons for refusal and hesitancy of the COVID-19 vaccination were also summarized by occupation types of participants and the survey rounds. Data analysis was processed using Stata 15.0.

## 3. Results

### 3.1. Socio-Demographical Characteristics of the Sample

In the first survey, 1196 participants gave responses to the online questionnaire. Among them, 148 participants reported to be retired or unemployed, or did not provide their occupation in the survey, and one participant did not indicate the willingness to accept the COVID-19 vaccine. Therefore, there were 1047 valid respondents to the first survey. For the second survey, a total of 1000 participants responded (30% response rate) and provided valid responses to the research questions. In the first survey (Table 1), 67.9% were female, and 20.7% of them were aged 18–29, 28.1% aged 30–39, 33.0% aged 40–49 and 18.3% aged 50 and above. The majority of them (75.1%) had received an education at university or achieved a higher academic degree. Over half of them (53.1%) were married or cohabited. Most participants (59.5%) were professionals or managers and administrators, while only 1.2% of them were blue-collar workers. There were 14.4% of them who had chronic conditions and 18.4% who had received the influenza vaccine in 2019. In the second survey, 46.1% were female, and 16.1% aged 18–29, 22.5% aged 30–39, 24.7% aged 40–49 and 36.7% aged 50 and above. Among them, 70.0% had finished preparatory school or a higher degree, and 57.2% were married or cohabited. Over half of them (51.6%) were clerical support, service and sales workers or blue-collar workers. In this survey, 10.8% of them had chronic conditions, and 17.7% had received the influenza vaccine in 2019.

### 3.2. Willingness to Accept the COVID-19 Vaccine

**Figure 1:**
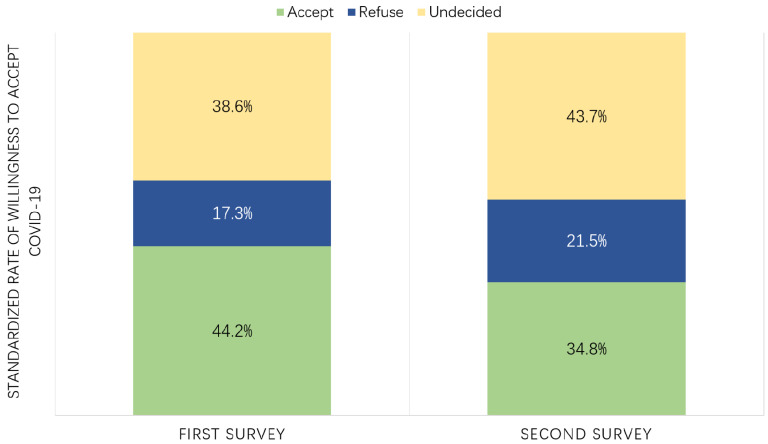
Difference in standardized rate of willingness to accept COVID-19 between two surveys.

Standardized rate of the vaccine acceptance in the first survey was calculated based on distribution of the second survey to make the two surveys comparable. Working people were found to be more willing to accept the vaccine (44.2% vs. 34.8%) and less likely to be hesitant (38.6% vs. 43.7%) in the first survey than those in the second one (*p* < 0.001) (Table 2 and Figure 1).

From the univariate analysis (Table 1), the willingness of the participants to accept the COVID-19 vaccine was found to be associated with age (*p* < 0.001), sex (*p* < 0.001) and previous influenza vaccine uptake (*p* < 0.001) in the first survey, while marriage (*p* < 0.001), occupation (*p* = 0.044), chronic condition (*p* = 0.026) and previous influenza vaccine uptake (*p* < 0.001) were identified in the second survey. These factors, except education, were used as covariates in multiple regressions examining a change in willingness of participants to accept the vaccine between the two surveys, as inclusion of education would result in multicollinearity. In the multiple multinomial logistic regression (Table 3), compared to those with hesitancy, working people were less likely to accept the COVID-19 vaccine in the second survey than those in the first one (adjusted odds ratio (AOR): 0.68, 95% confidence interval (CI): 0.56–0.84), while no significant difference was found for people who refused the vaccine (AOR: 1.12, 95% CI: 0.88–1.44).

In addition to the difference between the time points of the two surveys, clerical, service or sales workers were less likely to accept the vaccine (AOR: 0.62, 95%CI: 0.43–0.91), while blue-collar workers were less likely to refuse it (AOR: 0.52, 95% CI: 0.32–0.86). Those who received the influenza vaccine in the past year were more likely to accept the vaccine (AOR: 2.25, 95% CI: 1.74–2.93) and less likely to refuse it (AOR: 0.42, 95% CI: 0.27–0.66). Moreover, those with advanced age were less likely to accept the vaccine and more likely to refuse it. Female participants were also less likely to accept it.

### 3.3. Participant Reasons for COVID-19 Vaccine Refusal or Hesitancy

**Figure 2:**
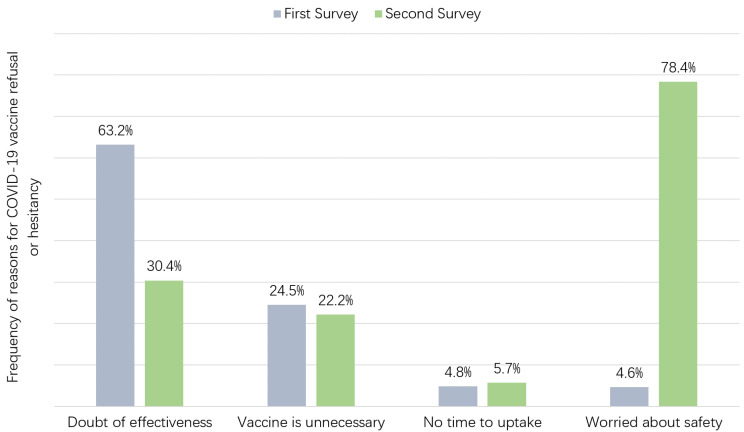
Reasons for COVID-19 vaccine refusal or hesitancy in two surveys.

The participants who were not willing to accept the vaccine (i.e., vaccine refusal) or were unsure (i.e., vaccine hesitancy) were asked about their reasons for refusal or hesitancy (Table 4 and Figure 2). In the first survey, 63.2% of the participants had doubts in the effectiveness of the vaccine, 24.5% of them believed the vaccine was unnecessary, 4.8% indicated lack of time and 4.6% worried about the safety or side effects of the vaccination. Significant differences among occupations were found in the belief that vaccination is unnecessary (*p* = 0.031) and worries about safety (*p* = 0.012). Clerical/service/sales and blue-collar workers tended to have a lower level of these two concerns. In the second survey, there were more participants who worried about the safety issue of the vaccine (78.4%), while less of them worried about the effectiveness (30.4%). There were still 22.2% who believed the vaccine was unnecessary and 5.7% who reported a lack of time to uptake the vaccine. Among occupations, more clerical, service and sales workers (27.2%) and blue-collar workers (26.3%) tended to believe the vaccine was unnecessary than others (*p* = 0.036), and more blue-collar workers reported that lack of time was one of their reasons for vaccine refusal or hesitancy (12.0%, *p* = 0.007).

### 3.4. Personal Protection Behaviours to Prevent Infection

**Figure 3:**
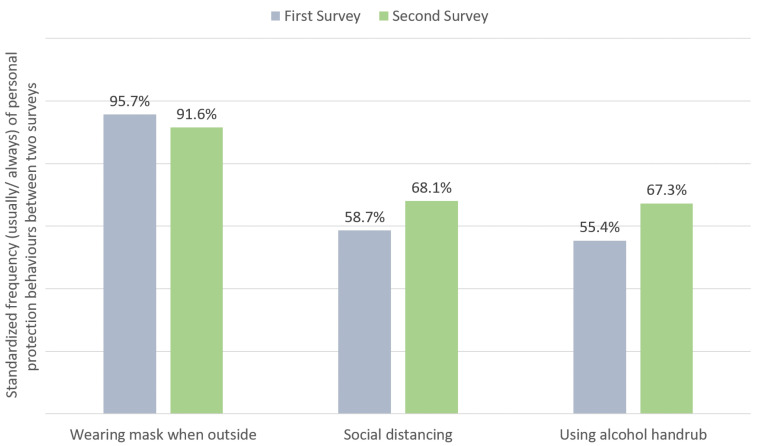
Difference in standardized frequency (usually/always) of personal protection behaviours between two surveys.

In addition to the COVID-19 vaccine, associations of personal protection behaviours such as frequency of wearing a facemask when outside, maintaining social distancing and use of alcohol hand rub during the two survey time points as well as other factors were examined. Compared with the first survey, fewer people in the second survey maintained their frequency of wearing facemask (91.6% vs. 95.7%, *p* < 0.001), but more of them expressed maintaining social distancing (68.1% vs. 58.7%, *p* = 0.001) and applying alcohol hand rub (67.3% vs. 55.6%, *p* < 0.001) more often (Table 2 and Figure 3).

In the multiple regressions (Table 3), it was found that participants were less likely to usually or always wear a facemask outdoors in the second survey than the first one (AOR: 0.47, 95% CI: 0.32–0.69), while more likely to maintain social distancing (AOR: 1.54, 95% CI: 1.27–1.86) and use alcohol hand rub (AOR: 1.67, 95% CI: 1.39–2.02). Blue-collar workers were less likely to maintain social distancing (AOR: 0.54, 95% CI: 0.37–0.77) but more likely to use hand rub when outside (AOR: 1.47, 95% CI: 1.03–2.10). Managers and administrators were also more likely to use the alcohol hand rub (AOR: 2.08, 95% CI: 1.41–3.07). Despite this, working people with advanced age were less likely apply alcohol hand rub for prevention of COVID-19.

## 4. Discussion

To our knowledge, this is among the first studies to investigate the trend of acceptance of the COVID-19 vaccine during the pandemic. The two surveys in this study were conducted at the first wave of local epidemic before declaration of the COVID-19 pandemic and at the third local wave after the pandemic declaration, separately [26]. During the 11 days of the first survey, a total of 35 confirmed cases were identified in HK with daily increments of fewer than 10, while there were 207 confirmed cases during the 15 day second survey with daily increments ranging from 7 to 24 [25]. Although the local situation was more severe during the second survey, a reduction in willingness to accept the COVID-19 vaccine and a higher level of vaccine hesitancy among the working population in HK were found. The acceptance rate of the vaccine in the second survey was 34.5%, which was fairly low to achieve herd immunity with at least 60% coverage of a vaccine with 100% efficacy for life-long protection [31]. The findings remind us that more efforts are needed to promote the COVID-19 vaccination.

According to the reasons for vaccine refusal and hesitancy reported by the participants, more people had doubts or concerns over the effectiveness and safety of the potential vaccine than believing it unnecessary or having no time to uptake it. This result was similar to the finding reported in a previous study in HK during the 2009 H1N1 pandemic that worries about side effects were more common than other reasons for rejection of the vaccine among health care workers [32]. The study in China found that although over 90% of the respondents stated that they would accept the COVID-19 vaccine when available, almost 50% of these people wanted to delay the vaccination until it was confirmed safe [17]. It is also similar to a survey in Canada which reported that major concerns of people who reported they were unlikely to accept the COVID-19 vaccine were risks, safety and side effects of vaccination [15]. Another study in Malaysia reported that over 95% of the respondents had concerns about the efficacy, effectiveness and safety of the COVID-19 vaccine [18]. In addition to a higher level of concern over safety and side effects of the vaccine than other reasons, this concern was found to be higher in the second survey than the first one, suggesting that the safety of the vaccine could be among the major reasons why fewer people were willing to accept it, while more people reported hesitancy. This change may not only be attributed to reasonable concerns based on inconclusive clinical trial results on safety of the vaccines, which can be alleviated with scientific evidence when the trials are finished, but could also result from diminished vaccine confidence enhanced by online misinformation over time [10,33,34,35,36], which may eventually cause refusal of the vaccines even if they are proven to be effective and safe.

Meanwhile, the frequencies of personal protection behaviours changed between the survey time points. Slightly fewer people were found to frequently wear masks outdoors in the second survey, but the overall rate of mask-wearing remained at a high level (over 90% of people). There were more working people who had a higher compliance with social distancing and use of alcohol hand rub when outside in the second survey, which showed a growing awareness and positive attitude towards these individual-level precautions [37,38]. Taking both increasing compliance to social distancing and hand rub usage and decreasing vaccine acceptance rate into consideration, the findings implied that the participants perceived their good personal protection behaviours as substitutes for vaccination to prevent COVID-19. The general public might believe these precautions would be sufficient to prevent COVID-19 without receiving the vaccine based on their personal experience of recent months during the pandemic. A similar finding was reported in a study among health care workers in Canada, which found that those who did not received the influenza vaccination tended to believe that preventive measures other than vaccination, including hand-washing and exercising, were more effective than vaccination [39]. Thus, the decreasing trend of vaccination acceptance rate might partially result from an increasing positive perception on these individual infection control precautions.

Considering all this, information dissemination efforts on safety of the vaccination, which are as important as efficacy and effectiveness of the vaccine, should be enhanced by health authorities and organizations, particularly through online social media where the issue of misinformation is severe [35,36,40]. The primary care sector should also be engaged in health education to increase vaccination coverage [41]. For the information dissemination, there should not only be information about the safety of the vaccination but also messages conveying that vaccination is a part of the individual infection control precautions that would complement social distancing and handwashing in disease prevention, and could be helpful to both individuals and communities to resume normal life. On the other hand, despite the urgent situation of the pandemic, the development of the COVID-19 vaccine should be rushed without proper and thorough examination, as this could cause adverse events more easily and would subsequently jeopardize the public’s trust in scientific communities, manufacturers, health authorities and the vaccine itself, and lead to a reduction in uptake rate of the vaccine eventually [10].

Differences in vaccination acceptance were found among different occupations, which may be attributed to differences in knowledge of and attitude to prevention precautions among occupations [42]. The clerical, sales and service workers had a lower willingness to accept the COVID-19 vaccine than the others, which is similar to findings from the US that influenza vaccination coverage was lower in sales and service workers than most occupations [43]. They were also more likely to believe that the vaccination is unnecessary, which might result from a lower health literacy or the belief that the threats of the pandemic were exaggerated [16]. However, these workers, including receptionists, attendants and salespersons, had a greater frequency of contact with other people due to the nature of their work and, therefore, were exposed to greater risks of COVID-19 infection [21,23]. They also have longer working hours but receive lower salaries than professionals and managers/administrators, so they were more likely to have lower socio-economic status [27]. In light of this, interventions and policies to improve and facilitate the vaccination of people with these occupations should be considered. These workers who have frequent exposure to crowds should be included as one of the priority groups in addition to residents of care homes, the elderly aged 65 or above, patients with chronic conditions and health care workers who are usually considered to be the priority groups of the seasonal influenza vaccination [44]. Health education in the workplace, convenience of vaccination and financial subsidies for the vaccination can be offered to these people to increase their uptake rate, and the involvement of their employers in these efforts is necessary.

Limitations in this study should be highlighted. First, this study comprises two cross-sectional surveys which may have heterogeneity between characteristics of the two survey samples. To minimize its influence on the results, standardization of the study sample was performed to make the two samples comparable, and multivariate analysis was used to adjust the influence of socio-demographical factors. Second, selection bias could exist due to the online data collection method. Nevertheless, the sample characteristics of the second survey matched the working population profile in HK, and the results from the first survey were standardized based on the sample of the second survey. Third, the study findings on change of acceptance rate of the vaccine could be influenced by local number of daily confirmed cases, capacity of healthcare services and relevant policies in different areas. Precautions should be taken to generalize the findings into other countries and regions. Moreover, the salaries of the participants were not collected in the surveys, which could be important in showing differences in vaccination acceptance among people with different socioeconomic status. However, the occupation types of the participants were collected in the studies and considered as a proxy for the socioeconomic factors associated with vaccination acceptance in the study. Trust in the government was also found as an important factor that is associated with acceptance of the COVID-19 vaccination in a study [45], which was not included as a covariate in this study. The influence of individual political views and trust in certain health authorities on uptake of the vaccination could be explored in future studies.

## 5. Conclusions

This study found a decreasing trend of the willingness to accept the COVID-19 vaccine between two local waves of the epidemic, which could be associated with growing confidence and compliance of personal protection behaviours. This implies that personal protection behaviours might be considered as substitutes of vaccination in prevention of the disease by working people. It seems that they tended to believe committing to these precautions should be sufficient for the COVID-19 prevention. This decreasing trend might also be an outcome of a high level of concern over vaccine safety. Future promotion of the vaccination should address these concerns, and a properly and thoroughly tested vaccine would be helpful to gain the confidence of the public. In addition, acceptance of the COVID-19 vaccine was lower among clerical/service/sales workers than the other occupations, although some of them are exposed to higher risks of infection due to the nature of their work. Policies that assist and improve the vaccine uptake of these workers should be considered. We believe it is important and more effective to start promotion, initiate policy-making and set up priority guidelines for the vaccination before the vaccines are approved, and this study could be used to inform these efforts. Further quantitative and qualitative studies could be conducted to follow up individuals for their vaccination acceptance and their reasons at different time points.

## Figures and Tables

**Table 1:** Socio-demographical characteristics of participants by willingness to accept COVID-19 vaccine in two surveys.

Characteristics	First Survey: Willingness to Accept COVID-19 Vaccine									Second Survey: Willingness to Accept COVID-19 Vaccine								
	Accept		Refuse		Undecided		*p* Value ^1^	Total		Accept		Refuse		Undecided		*p* Value ^1^	Total	
	N	%	N	%	N	%		N	%	N	%	N	%	N	%		N	%
Age																		
18–29	110	26.0	20	9.6	87	20.9	<0.001 *	217	20.7	53	15.2	27	12.6	81	18.5	0.063	161	16.1
30–39	125	29.6	55	26.4	114	27.4		294	28.1	82	23.6	53	24.7	90	20.6		225	22.5
40–49	125	29.6	82	39.4	138	33.2		345	33.0	80	23.0	66	30.7	101	23.1		247	24.7
50–59	49	11.6	44	21.2	69	16.6		162	15.5	79	22.7	48	22.3	116	26.5		243	24.3
60+	14	3.3	7	3.4	8	1.9		29	2.8	54	15.5	21	9.8	49	11.2		124	12.4
Sex																		
Male	168	39.7	65	31.3	103	24.8	<0.001 *	336	32.1	205	58.9	108	50.2	226	51.7	0.063	539	53.9
Female	255	60.3	143	68.8	313	75.2		711	67.9	143	41.1	107	49.8	211	48.3		461	46.1
Education																		
Below high school	2	0.5	3	1.4	5	1.2	0.170	10	1.0	14	4.0	8	3.7	23	5.3	0.279	45	4.5
High school	35	8.3	27	13.0	41	9.9		103	9.8	87	25.0	68	31.6	100	22.9		255	25.5
Preparatory	51	12.1	30	14.4	67	16.1		148	14.1	81	23.3	51	23.7	114	26.1		246	24.6
University or above	335	79.2	148	71.2	303	72.8		786	75.1	166	47.7	88	40.9	200	45.8		454	45.4
Marriage																		
Unmarried	201	47.5	94	45.2	196	47.1	0.854	491	46.9	117	33.6	101	47.0	210	48.1	<0.001 *	428	42.8
Married/cohabit	222	52.5	114	54.8	220	52.9		556	53.1	231	66.4	114	53.0	227	52.0		572	57.2
Occupation																		
Professionals	173	40.9	99	47.6	168	40.4	0.298	440	42.0	50	14.4	17	7.9	36	8.2	0.044 *	103	10.3
Manager/administrator	75	17.7	38	18.3	70	16.8		183	17.5	48	13.8	24	11.2	54	12.4		126	12.6
Associate professional	115	27.2	40	19.2	100	24.0		255	24.4	80	23.0	58	27.0	117	26.8		255	25.5
Clerical/service/sales worker	55	13.0	30	14.4	72	17.3		157	15.0	88	25.3	72	33.5	141	32.3		301	30.1
Blue-collar worker	5	1.2	1	0.5	6	1.4		12	1.2	82	23.6	44	20.5	89	20.4		215	21.5
Chronic disease																		
No	363	85.8	179	86.1	354	85.1	0.934	896	85.6	298	85.6	194	90.2	400	91.5	0.026 *	892	89.2
Yes	60	14.2	29	13.9	62	14.9		151	14.4	50	14.4	21	9.8	37	8.5		108	10.8
Influenza vaccine uptake last year																		
No	309	73.1	190	91.4	355	85.3	<0.001 *	854	81.6	253	72.7	199	92.6	371	84.9	<0.001 *	823	82.3
Yes	114	27.0	18	8.7	61	14.7		193	18.4	95	27.3	16	7.4	66	15.1		177	17.7
Total	423	100.0	208	100.0	416	100.0		1047	100.0	348	100.0	215	100.0	437	100.0		1000	100

* *p* < 0.05. ^1^ *p* values are from the Chi-square test or Fisher’s exact tests.

**Table 2:** Difference in willingness to accept COVID-19 vaccine and frequency of personal protection behaviours between two surveys.

Acceptance of Vaccination/Behaviours	First Survey			Second Survey		*p* Value ^2^
	N	Unstandardized %	Standardized % ^1^	N	Standardized % ^1^	
Willingness to accept COVID-19 vaccine						
Accept	423	40.4	44.2	348	34.8	<0.001
Refuse	208	19.9	17.3	215	21.5	
Undecided	416	39.7	38.6	437	43.7	
Wearing mask when outside						
Never/sometimes	48	4.6	4.3	84	8.4	<0.001
Usually/always	999	95.4	95.7	916	91.6	
Social distancing						
Never/sometimes	343	32.8	41.3	319	31.9	0.001
Usually/always	704	67.2	58.7	681	68.1	
Using alcohol hand rub						
Never/sometimes	457	43.7	44.5	327	32.7	<0.001
Usually/always	590	56.4	55.6	673	67.3	
Total	1047	100.0	100.0	1000	100.0	

^1^ Standardized rate in the first survey was calculated based on age, sex and occupational distribution of sample in the second survey. Standardized rate in the second survey was the same as the unstandardized rates because the same sample was used as the reference population. ^2^ *p* values are from the Chi-square test.

**Table 3:** Changes of willingness to accept COVID-19 vaccine and frequency of personal protection behaviours between two surveys.

Characteristics	COVID-19 Vaccine Acceptance (Undecided as Reference) ^1^				Usually/Always Wear Mask When Outside^1^		Usually/Always Maintain Social Distance ^1^		Usually/Always Use Alcohol Hand Rub When Outside ^1^	
	Accept		Refuse							
	AOR ^2^	95%CI ^2^	AOR	95% CI	AOR	95% CI	AOR	95% CI	AOR	95% CI
Age										
18–29	(reference)		(reference)		(reference)		(reference)		(reference)	
30–39	0.67 *	(0.48, 0.93)	1.76 *	(1.10, 2.82)	0.81	(0.44, 1.49)	1.08	(0.79, 1.47)	0.91	(0.67, 1.25)
40–49	0.56 *	(0.39, 0.80)	3.04 *	(1.88, 4.91)	1.28	(0.64, 2.55)	0.87	(0.62, 1.21)	0.58 *	(0.42, 0.81)
50–59	0.37 *	(0.26, 0.53)	2.20 *	(1.36, 3.56)	1.26	(0.63, 2.51)	0.78	(0.56, 1.08)	0.41 *	(0.29, 0.57)
60+	0.70	(0.46, 1.08)	2.74 *	(1.55, 4.82)	0.66	(0.32, 1.36)	0.95	(0.64, 1.41)	0.58 *	(0.39, 0.85)
Sex										
Male	(reference)		(reference)		(reference)		(reference)		(reference)	
Female	0.71 *	(0.57, 0.89)	1.04	(0.80, 1.35)	1.40	(0.94, 2.09)	1.37 *	(1.11, 1.67)	1.60 *	(1.31, 1.97)
Marriage										
Unmarried	(reference)		(reference)		(reference)		(reference)		(reference)	
Married/cohabit	1.69 *	(1.33, 2.14)	0.75 *	(0.58, 0.98)	0.80	(0.52, 1.22)	1.49 *	(1.21, 1.85)	1.63 *	(1.32, 2.02)
Chronic disease										
No	(reference)		(reference)		(reference)		(reference)		(reference)	
Yes	1.07	(0.80, 1.44)	1.17	(0.82, 1.67)	0.56*	(0.35, 0.89)	0.88	(0.67, 1.14)	0.93	(0.72, 1.21)
Influenza vaccine uptake last year										
No	(reference)		(reference)		-		-		-	
Yes	2.25 *	(1.74, 2.93)	0.42 *	(0.27, 0.66)	-		-		-	
Occupation										
Professionals	(reference)		(reference)		(reference)		(reference)		(reference)	
Manager/administrator	0.81	(0.53, 1.25)	0.70	(0.42, 1.16)	0.79	(0.35, 1.81)	1.01	(0.68, 1.51)	2.08 *	(1.41, 3.07)
Associate professional	0.77	(0.53, 1.12)	0.82	(0.52, 1.29)	0.67	(0.33, 1.37)	0.95	(0.67, 1.34)	1.27	(0.91, 1.77)
Clerical/service/sales worker	0.62 *	(0.43, 0.91)	0.68	(0.44, 1.06)	0.70	(0.34, 1.44)	0.93	(0.66, 1.32)	1.36	(0.97, 1.89)
Blue-collar worker	0.76	(0.52, 1.13)	0.52 *	(0.32, 0.86)	0.69	(0.33, 1.45)	0.54 *	(0.37, 0.77)	1.47 *	(1.03, 2.10)
Wave of local epidemic										
First wave (first survey)	(reference)		(reference)		(reference)		(reference)		(reference)	
Third wave (second survey)	0.68 *	(0.56, 0.84)	1.12	(0.88, 1.44)	0.47 *	(0.32, 0.69)	1.54 *	(1.27, 1.86)	1.67 *	(1.39, 2.02)

* *p* < 0.05. ^1^ In these regressions, the sample of the first survey was adjusted based on age, sex and occupational distribution of the sample in the second survey. ^2^ AOR: adjusted odds ratio; CI: confidence interval.

**Table 4:** Reasons for COVID-19 vaccine refusal or hesitancy among different occupations in two surveys.

Occupations	Doubt of Effectiveness		Vaccine Is Unnecessary		No Time to Uptake		Worried About Safety		Total
	No	Yes	No	Yes	No	Yes	No	Yes	
First survey (standardized N (%)) ^1^									
Professional	25 (40.3)	37 (59.7)	43 (69.1)	19 (30.9)	56 (91.6)	5 (8.4)	59 (96.1)	2 (3.9)	62 (100)
Manager/administrator	35 (46.4)	41 (53.6)	50 (65.8)	26 (34.2)	76 (99.2)	1 (0.8)	69 (90.1)	8 (9.9)	76 (100)
Associate professional	44 (34.3)	85 (65.7)	95 (73.7)	34 (26.3)	123 (95.3)	6 (4.7)	120 (93.1)	9 (6.9)	129 (100)
Clerical/service/sale worker	79 (38.8)	125 (61.2)	147 (71.7)	58 (28.4)	189 (92.6)	15 (7.4)	198 (96.7)	7 (3.3)	205 (100)
Blue-collar worker	22 (25)	66 (75.1)	88 (100)	0 (0)	88 (100)	0 (0)	88 (100)	0 (0)	88 (100)
*p* value ^2^	0.058		0.031 *^,3^		0.300 ^3^		0.012 *^,3^		
Total	206 (36.8)	354 (63.2)	423 (75.5)	137 (24.5)	533 (95.2)	27 (4.8)	534 (95.4)	26 (4.6)	560 (100)
Second survey (N (%))									
Professional	38 (71.7)	15 (28.3)	43 (81.1)	10 (18.9)	50 (94.3)	3 (5.7)	11 (20.8)	42 (79.3)	53 (100.0)
Manager/administrator	50 (64.1)	28 (35.9)	68 (87.2)	10 (12.8)	73 (93.6)	5 (6.4)	15 (19.2)	63 (80.8)	78 (100.0)
Associate professional	118 (67.4)	57 (32.6)	143 (81.7)	32 (18.3)	169 (96.6)	6 (3.4)	29 (16.6)	146 (83.4)	175 (100.0)
Clerical/service/sale worker	150 (70.4)	63 (29.6)	155 (72.8)	58 (27.2)	206 (96.7)	7 (3.3)	53 (24.9)	160 (75.1)	213 (100.0)
Blue-collar worker	98 (73.7)	35 (26.3)	98 (73.7)	35 (26.3)	117 (88)	16 (12)	33 (24.8)	100 (75.2)	133 (100.0)
*p* value ^2^	0.604		0.036 *		0.007 *		0.282		
Total	454 (69.6)	198 (30.4)	507 (77.8)	145 (22.2)	615 (94.3)	37 (5.7)	141 (21.6)	511 (78.4)	652 (100)

* *p* < 0.05. ^1^ Count and proportions of the first survey were adjusted based on age, sex and occupational distribution of the sample in the second survey. Some numbers did not add up to a total number due to rounding of decimals. ^2^ *p* values are from the Chi-square test or Fisher’s exact tests. ^3^ Due to zero count in cells, these *p* values were calculated by combining clerical/service/sale workers and blue-collar workers into one group.

## Data Availability

Data used in this study cannot be made publicly available for ethical reasons. Public availability of data would compromise confidentiality and privacy of participants.
